# Constipation and diarrhoea - common adverse drug reactions? A cross sectional study in the general population

**DOI:** 10.1186/1472-6904-11-2

**Published:** 2011-02-18

**Authors:** Gunvor S Fosnes, Stian Lydersen, Per G Farup

**Affiliations:** 1 Department of Medicine, Innlandet Hospital Trust, Gjøvik, Norway; 2 Unit for Applied Clinical Research, Department of Cancer Research and Molecular Medicine, Norwegian University of Science and Technology, Trondheim, Norway

## Abstract

**Background:**

Constipation and diarrhoea are common complaints and often reported as adverse drug reactions. This study aimed at finding associations between drugs and constipation and diarrhoea in a general population.

**Methods:**

A selection of inhabitants in Oppland County, Norway participated in a cross-sectional survey. Information about demographics, diseases including gastrointestinal complaints classified according to the Rome II criteria and use of drugs were collected on questionnaires. Constipation was defined as functional constipation and constipation predominant Irritable Bowel Syndrome (IBS), and diarrhoea as functional diarrhoea and diarrhoea predominant IBS. Associations between drugs and constipation and diarrhoea were examined with multivariable logistic regression models. Based on the multivariable model, the changes in prevalence (risk difference) of the abdominal complaints for non-users and users of drugs were calculated.

**Results:**

In total 11078 subjects were invited, 4622 completed the questionnaires, 640 (13.8%) had constipation and 407 (8.8%) had diarrhoea. To start using drugs increased the prevalence of constipation and diarrhoea with 2.5% and 2.3% respectively. Polypharmacy was an additional risk factor for diarrhoea. Use of furosemide, levothyroxine sodium and ibuprofen was associated with constipation, and lithium and carbamazepine with diarrhoea. The excess drug related prevalence varied from 5.3% for the association between ibuprofen and constipation to 27.5% for the association between lithium and diarrhoea.

**Conclusions:**

Use of drugs was associated with constipation and diarrhoea in the general population. The associations are most likely adverse drug reactions and show that drug-induced symptoms need to be considered in subjects with these complaints.

## Background

Constipation and diarrhoea are worldwide complaints with some variations between geographical regions, populations and definitions [1-7]. The use of drugs is common, increases with age and has increased over time with a doubling of prescriptions to elderly from 1996 to 2006 [8,9]. Unfortunately, drugs are associated with adverse drug reactions (ADRs) which increase with polypharmacy [3,10-16]. Gastrointestinal complaints like constipation and diarrhoea are common ADRs [16-18]. The reported prevalence rates of gastrointestinal ADRs, especially constipation and diarrhoea, are based mainly on clinical drug trials and observational studies in selected, often elderly, populations, and use heterogeneous definitions of constipation and diarrhoea [3,11,14,17,18]. The prevalence of constipation and diarrhoea related to everyday use of drugs in an unselected general population is in large unknown.

Increased knowledge about ADRs allows individual adjustment of drug treatment in patients with gastrointestinal symptoms. This population based cross-sectional study aimed at finding associations between drugs and constipation and diarrhoea.

## Methods

### Participants

In 2001, all persons in Oppland County, Norway, born in 1970, 1960, 1955, 1940 and 1925, were invited to a health study conducted by the Norwegian Institute of Public Health who also collected the data [19].

### Design

The study was population-based with a cross-sectional design. No remuneration was given. At attendance, standardized questionnaires were completed and a physical examination was performed. All subjects were asked to take home, complete and return by post a supplementary questionnaire about the complaints. Non-responders received two reminders.

### Variables

#### Questionnaires

All participants filled in detailed questionnaires with information about age, gender, height, weight, cohabiting, years of education, physical activity (rated less than 1 hour/week, 1-2 hours/week and more than 3 hours/week), number of cups of coffee per day, use of alcohol (8 point ordinal scale from 1 = "never" to 8 = "4-7 times/week") and smoking habits (never, past, current). All present and previous diseases were noted. Musculoskeletal complaints the last four weeks were assessed for six locations (neck/shoulder, arms/hands, upper back, lower back, hip/legs/feet and other locations) and the intensity for each of them was rated as none, mild or severe. A musculoskeletal complaints score was calculated by multiplying number of locations and intensity giving a score with range 0-12. Mood disorders (mainly anxiety and depression) were measured with Hopkins' Symptom Check List-10 (HSCL-10) with range 1.0-4.0 and 1.85 as upper normal limit [20]. The subjects gave a written report on all drugs used regularly the last four weeks. The questionnaires have been translated into Norwegian, validated and extensively used in national surveys by the Norwegian Institute of Public Health [19]. Gastrointestinal complaints were assessed with a questionnaire based on the Rome II criteria for functional gastrointestinal disorders [21]. This survey has previously been used for the study of the prevalence, co-morbidity and impact of irritable bowel syndrome [22].

#### Definition of constipation and diarrhoea

The gastrointestinal symptom questionnaire allowed classification of the disorders into functional constipation, functional diarrhoea and Irritable Bowel Syndrome (IBS) with the subgroups diarrhoea predominant, constipation predominant and alternating, in accordance with the Rome II criteria [21]. In this study, constipation includes functional constipation and constipation predominant IBS, and diarrhoea includes functional diarrhoea and diarrhoea predominant IBS.

#### Classification of drugs

Drug substances were classified according to the Anatomical-Therapeutic-Chemical Classification System (ATC) at level 5 and as group of drugs at ATC level 4 [23]. Use of drugs was measured as yes/no (Yes = use of one or more drugs), as number of drugs and on a categorical scale (0 = no drugs, 1 = use of one drug, 2 = use of 2-3 drugs, and 3 = use of >3 drugs). Polypharmacy was defined as using more than three drugs.

Analyses were performed for individual drugs (ATC-level 5) and groups of drugs at ATC level 4, which are rather similar and probably have concurrent ADRs. However, no analyses were performed for drugs used for gastrointestinal disorders (ATC-classes A02, A03, A06 and A07) since drug related gastrointestinal symptoms could not be distinguished from the disorder under treatment. Nor were drugs or groups of drugs used by less than 10 persons analysed.

### Statistics

Bivariate analyses of the association between the abdominal complaint and one variable at a time were analysed with Student's t-test, Wilcoxon-Mann-Whitney's test, or Fisher's exact test (Tables 1 and 2). In multivariable logistic regression models with abdominal complaint as outcome, the effects of drugs were adjusted for covariates selected as follows: Candidate covariates were all background variables listed in Table 1. Forward likelihood ratio (LR) selection with p-enter = 0.15 and p-remove = 0.20 was followed by backwards LR with p-enter = 0.05 and p-remove = 0.06, a procedure adapted from Hosmer and Lemeshow [24]. Age and gender were included in all models. Separate analyses were performed for drug use no/yes (Table 3), number of drugs (Table 4), and drug substances (Table 2). Candidate drug substances (for Table 5) were those with unadjusted p-values ≤ 0.2 and groups of drugs with p-value ≤0.2 if none of the substances in the group were candidates. Based on the multivariable model, we estimated the average change in prevalence (also called average risk difference) of the abdominal complaint for non-users and users of drugs and the separate drug substances, if they would start or stop using the drugs [25]. Unlike in randomized controlled trials, the characteristics of users and non-users of the drugs differ. Hence, the effect of the drug in terms of risk difference is not the same among users and non-users of the drug. The two risk differences are estimated using the method of Bender et al [26].

**Table 1:** Characteristics of all participants (independent of complaints), participants with constipations and participants with diarrhoea, and comparisons between subjects with and without diarrhoea and with and without constipation.

Characteristics	All participants n = 4622	Participants with constipation n = 640	Participants with diarrhoea n = 407
Age (years)	49.4 (13.8)	52.2 (15.1)***	50.7 (13.7)†
Gender (male) (%)	43.6	21.3***	50.4**
Body mass index (kg/m^2^) [0.2%]	26.9 (4.2)	26.1(4.1)***	27.2 (4.1)*
Cohabiting (%) [14.9%]	89.3	87.9	86.2*
Years of education [1.0%]	12.2 (3.5)	12.0 (3.5) †	11.7 (3.4) *
Physical activity (breathless) (%) [7.2%] <1 hr/1-2 hrs/>3 hrs a week	58.8/27.0/14.2	60.9/27.7/11.4 *	60.6/26.9/12.5
Cups of coffee/day [1.0%]	3.7 (2.8)	3.4 (2.6) *	3.8 (2.9)
Frequency of use of alcohol ¤ [0.8%]	4.4 (1.7)	4.1 *** (1.7)	4.6 * (1.7)
Use of alcohol >1 time/week (%)	10.6	8.2 *	12.9 †
Smoking habits (%) [0.6%] Never/Past/Current	44.5/27.2/28.3	46.1/27.4/26.5	35.9/31.4/32.7 ***
Asthma (%) [2.1%]	8.7	10.6 *	9.8
Allergic rhinitis (%) [15.4%]	12.9	13.6	13.5
Bronchitis (%) [3.5%]	2.5	2.7	2.5
Diabetes (%) [2.5%]	2.1	3.3 *	3.4 †
Osteoporosis (%) [3.3%]	2.4	4.7 ***	3.7 †
Myocardial infarction (%) [2.4%]	2.8	2.8	3.7
Angina pectoris (%) [2.4%]	3.7	6.1 *	4.2
Cerebral stroke (%) [2.5%]	2.0	3.1 *	3.7 *
Epilepsy (%) [0.8%]	1.6	2.4 †	1.7
Multiple sclerosis (%) [4.0%]	0.5	1.5 **	0.7
Musculoskeletal complaints §	2.2 (2.2)	2.6 (2.4) ***	2.7 (2.4) ***
HSCL 10 # [6.4%]	1.3 (0.4)	1.4 (0.5) ***	1.4 (0.5) ***
Use of drugs (Yes) (%)	62.5	72,5 ***	70.3 ***
Number of drugs	1.34 (1.56)	1.64 (1.67) ***	1.70 (1.78)***
Number of drugs (%) 0 drug/1 drug/2 or 3 drugs/≥4	37.5/28.3/24.5/9.8	27.5/30.3/29.7/12.5***	29.7/29.0/25.8/15.5***

The results are given as number of subjects, proportion (in percentage) or mean (with SD in brackets) and the proportion of missing data to each question in square brackets.

† = p < 0.20; * = p < 0.05; ** = p < 0.01; *** = p < 0.001.

¤ Frequency of use of alcohol: 8 point ordinal scale: 1 = never used to 8 = 4-7 times a week

§ Musculoskeletal complaints: Sum score (six location and intensity) 0-12

# HSCL 10: Hopkins Symptom Check List 10 (Mood disorders) 1-4

**Table 2:** The prevalence of constipation and diarrhoea among users and non-users of drugs significantly associated with the complaints (bivariate analyses, all participants)

	ATC level	ATC code	Name of drug group/chemical substance	Prevalence (%) Users/non users	Statistics (p-value)
Constipation	Level 4	C10AA	HMG CoA reductase inhibitors	18.5/13.6	0.033
		M01AE	Propionic acid derivatives	18.7/13.3	0.001
		N06AA	Non-selective monoamine reuptake inhibitors	23.2/13.7	0.050
	Level 5	C01AA04	Digitoxin	38.5/13.8	0.025
		C01DA02	Glyceryl trinitrate	27.3/13.7	0.015
		C03CA01	Furosemide	29.2/13.6	0.001
		C10AA05	Atorvastatin	21.4/13.7	0.037
		G03CX01	Tibolone	40.0/13.8	0.038
		H03AA01	Levothyroxine sodium	28.1/13.4	<0.001
		M01AE01	Ibuprofen	19.4/13.3	0.001
Diarrhoea	Level 4	C10AA	HMG CoA reductase inhibitors	12.7/8.6	0.031
		R06AD	Phenothiazine derivatives	28.6/8.7	0.029
	Level 5	A10BA02	Metformin	30.8/8.7	0.022
		C03EA01	Hydrochlorothiazide and potassium-sparing agents	25.0/8.7	0.009
		C07AB02	Metoprolol	13.3/8.6	0.026
		N03AF01	Carbamazepine	25.0/8.7	0.009
		N05AN01	Lithium	33.3/8.7	0.017
		N06AB05	Paroxetine	19.6/8.7	0.012
		R03BA01	Beclometasone	28.6/8.7	0.029

**Table 3:** Independent predictors for constipation and diarrhoea

	Independent predictor	OR		
		
		Estimates	95% CI	p-value
Constipation	Use of drug	1.30	1.06 to 1.59	0.012
	Gender (women)	3.24	2.61 to 4.02	<0.001
	Age (years)	1.01	1.003 to 1.02	0.005
	BMI (body mass index)	0.95	0.93 to 0.97	<0.001
	Frequency of use of alcohol	0.94	0.89 to 0.99	0.024
	Musculoskeletal complaints	1.04	1.002 to 1.09	0.042
	Angina pectoris	1.86	1.21 to 2.85	0.004
	Multiple sclerosis	2.41	1.03 to 5.66	0.043
				
Diarrhoea	Use of drugs	1.37	1.08 to 1.74	0.011
	Gender (Women)	0.69	0.55 to 0.86	0.001
	Age (years)	0.999	0.99 to 1.01	0.824 (ns)
	Frequency of use of alcohol	1.10	1.03 to 1.17	0.006
	Years of education/schooling	0.96	0.93 to 0.995	0.023
	HSCL 10 (Mood disorders)	1.70	1.37 to 2.11	<0.001
	Osteoporosis	2.20	1.21 to 3.97	0.009

Multivariable logistic regression analyses with stepwise selection of variables. Use of drugs and not the individual drugs were included in the models. Age and gender were included in the models independent of significance.

**Table 4:** Number of drugs as predictors for constipation and diarrhoea

	Number of drugs	OR		
		
		Estimates	95% CI	p-value
Constipation	None	1.00		
	Using one drug	1.34	1.07 to 1.69	0.012
	Using two or three drugs	1.26	0.99 to 1.61	0.062
	Using four or more drugs	1.21	0.85 to 1.71	0.288
				
Diarrhoea	None	1.00		
	Using one drug	1.31	0.995 to 1.73	0.055
	Using two or three drugs	1.30	0.96 to 1.75	0.087
	Using four or more drugs	1.91	1.31 to 2.78	0.001

Multivariable logistic regression analyses - adjusted for significant predictors.

Significant predictors for constipation: Age, gender, BMI, frequency of use of alcohol, musculoskeletal complaints, angina and multiple sclerosis.

Significant predictors for diarrhoea: Age, gender, frequency of use of alcohol, years of education, mood disorders and osteoporosis.

**Table 5:** Observed prevalence (in per cent) of constipation and diarrhoea in users and non-users of drugs, calculated prevalence if treatment is stopped or started, and changes of prevalence (average risk difference) with 95% CI when stopping and starting treatment (multivariable analyses, 4586 and 4268 cases available for the analyses of constipation and diarrhoea respectively)

Abdominal complaint	Predictor	Observed prevalence in nonusers	Calculated prevalence if starting treatment	Increased prevalence (95% CI)	Observed prevalence in users	Calculated prevalence if stopping treatment	Reduced prevalence (95% CI)
Constipation	Use of drugs	10.2	12.7	2.5	15.9	12.8	3.0
				(0.6 to 4.4)			(0.7 to 5.3)
	Furosemide	13.5	24.3	10.7	28.6	16.5	12.1
				(0.0 to 21.5)			(0.6 to 23.6)
	Levothyroxine sodium	13.3	18.7	5.4	26.8	19.5	7.3
				(-0.2 to 11.1)			(0.0 to 14.6)
	Ibuprofen	13.1	18.4	5.3	19.8	14.0	5.8
				(1.6 to 9.1)			(1.7 to 9.8)
Diarrhoea	Use of drugs	7.1	9.5	2.3	10.2	7.7	2.5
				(0.6 to 4.1)			(0.7 to 4.3)
	Lithium	8.9	36.2	27.2	36.4	8.9	27.5
				(-0.7 to 55.1)			(-0.6 to 55.5)
	Carbamazepine	8.9	27.7	18.8	28.6	9.4	19.2
				(-0.1 to 37.7)			(0.3 to 38.1)

Two-sided p-values ≤ 0.05 were regarded as statistically significant. Due to hypotheses testing for many substances, p-values above 0.01 should be interpreted with caution. We report 95% confidence intervals (CI) where appropriate. The analyses were performed in SPSS 16. The SPSS code "nne_ein.sps" available at Ralf Bender's software page was used for calculating change in prevalence [26].

### Ethics

This survey was approved by the Regional Committee for Medical Research Ethics and the Data Inspectorate, Oslo, Norway, and performed in accordance with the Declaration of Helsinki.

## Results

### Participants

Out of 11078 subjects invited to the health survey, 6141 participated. In the participants and non-participants the proportions of women was 55% and 45% respectively, and mean age was 49.2 and 45.0 years respectively.

**Figure 1:**
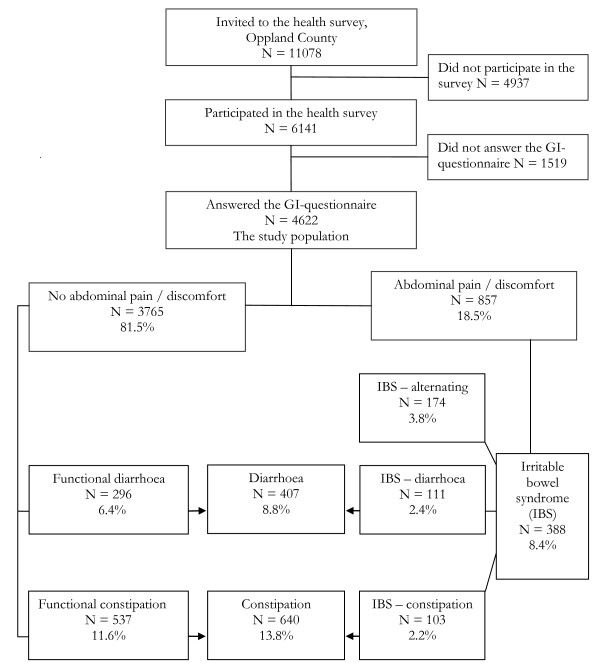
**Flow chart of the participants in the study with indication of complaints**. The study focuses on the groups with constipation (N = 640) and diarrhoea (N = 407).

The gastrointestinal questionnaire was completed by 4622 of the participants, 640 (13.8%) had constipation and 407 (8.8%) had diarrhoea. Two patients had both constipation and diarrhoea (diarrhoea was probably induced by use of laxatives). The responders were more likely to be women than the non-responders were (56% and 51% respectively), and younger (mean age 48.9 and 50.1 years respectively). Figure 1 shows the subjects in the study and the classification of functional gastrointestinal disorders in more detail. Table 1 presents the characteristics of all participants and of participants with constipation and diarrhoea, and gives comparisons between those with and without constipation and with and without diarrhoea. Age, gender, body mass index, use of alcohol, osteoporosis, musculoskeletal complaints, mood disorders, use of drugs and number of drugs were highly significantly associated with constipation; and smoking, musculoskeletal complaints, mood disorders, use of drugs and number of drugs with diarrhoea.

### Use of drugs and associations with constipation and diarrhoea

In all, 288 different generic drugs were used, 252 after exclusion of drugs for gastrointestinal disorders. After exclusion of drugs and groups of drugs used by <10 persons, 98 substances (ATC-level 5) and 20 groups at ATC-level 4 were included in the analyses.

The mean number of drugs per person was 1.34 (range 0-9), and 2891 (62.5%) used one or more drugs. Use of drugs was associated with constipation and diarrhoea, the unadjusted ORs were 1.69 and 1.46 respectively (p ≤ 0.001), and likewise, adjusted OR were 1.30 (p = 0.012) and 1.37 (p = 0.011). Table 3 gives independent predictors of constipation and diarrhoea.

Table 4 shows the association between numbers of drugs and constipation and diarrhoea. Using more than one drug was not associated with constipation over that of one drug alone. However, polypharmacy increased the prevalence of diarrhoea significantly, compared with using one drug and 2-3 drugs the ORs were 1.46 (CI 1.01 to 2.10, p = 0.042), and 1.47 (CI 1.03 to 2.11, p = 0.034) respectively.

### Drug substances associated with constipation and diarrhoea

Drugs and group of drugs associated with constipation and diarrhoea with p≤0.05 in bivariate analyses are listed in table 2. In addition, 15 and 12 drugs were associated with constipations and diarrhoea respectively with p≤0.20. Three drugs were independent predictors of constipation: furosemide (OR 2.16, CI 1.14 to 4.10, p = 0.019), levothyroxine sodium (OR 1.55, CI 1.04 to 2.31, p = 0.033) and ibuprofen (OR 1.54, CI 1.17 to 2.03, p = 0.002), and two with diarrhoea: lithium (OR 6.09, CI 1.73 to 21.48, p = 0.005) and carbamazepine (OR 4.07, CI 1.52 to 10.89, p = 0.005) (logistic regression analyses with correction for the independent predictors given in table 3). No groups of drugs at ATC-level 4 were independent predictors of constipations or diarrhoea without one substance in the group being associated with the disorders.

Table 5 gives the observed prevalence of constipation and diarrhoea for users and nonusers of drugs and for drugs significantly associated to the complaint, the calculated prevalence if users stop and nonusers start treatment and the change in prevalence when stopping and starting treatment. To start using drugs would increase the prevalence of constipation and diarrhoea with 2.5% and 2.3% respectively. The excess drug related prevalence varied from 5.3% for the association between ibuprofen and constipation, to 27.5% for the association between lithium and diarrhoea.

## Discussion

Use of drugs was significantly associated with constipation and diarrhoea in this study in the general population, as it has been in studies performed in general practice, in elderly and in other settings [3,7,14,16,18]. The prevalence of drug associated constipation and diarrhoea was judged as high (2.3% - 3.0%), particularly because compliance declines markedly in subjects with ADRs [27]. It is to expect that subjects with ADRs reduce or stop treatment, or switch to alternatives to avoid the complaints, and thereby reduce the prevalence of ADRs. However, it is likely that some subjects are unaware of the connection between the complaints and the drug. The results indicate an unfavourable effect of everyday use of drugs on constipation and diarrhoea in the general population. Changes in drug therapy might be the most important easily susceptible factor to influence on in subjects with these disorders.

Polypharmacy is a risk factor for ADRs in general, and especially among the elderly [3,10-15]. This study showed, however, only an association between polypharmacy and diarrhoea. One possible explanation of the difference between constipation and diarrhoea is that more drugs are associated with diarrhoea than with constipation.

As expected, several drugs were associated with constipation and diarrhoea in bivariate analyses (table 2), but the number of specific drugs associated with constipation and diarrhoea in multivariable analyses was lower than expected: three and two respectively. Constipation has been mentioned as an ADR to both furosemide and ibuprofen in some reports and high quality information about marketed drugs [7,16,28]. The pathophysiology is uncertain. Dehydration might cause constipation in users of furosemide, and inhibition of prostaglandins might explain constipation in ibuprofen users since prostaglandin analogues cause diarrhoea [29]. Diarrhoea has been related to too high doses of levothyroxine sodium, constipation has not [28]. Since severe hypothyroidism is associated with constipation, the association between levothyroxine sodium and constipation might have been confounded by the disorder under treatment (hypothyroidism) or insufficient treatment, or it might be a type I error. Carbamazepine and lithium were both associated with diarrhoea, which is a known ADR to these drugs [28]. The excess prevalence in users was surprisingly high (19 and 27% respectively), particularly for lithium because gastrointestinal ADRs are expected to level off and/or decline with continuous use [30]. The fact that it might be undesirable to change lithium therapy if the effect is satisfactory could explain the high ADR rate. In all, the excess prevalence of constipation and diarrhoea associated with these five drugs was high (5-27%) related to the authorities definition of an ADR as common if the prevalence is between 1-10% [31]. The Rome II definition of constipation and diarrhoea includes mild and intermittent symptoms, which might be missed in clinical trials. Therefore, the clinical relevance of the findings is uncertain, but calls attention to these drugs in patients bothered by constipation and diarrhoea. The overall prevalence of constipation and diarrhoea in this study (13.8% and 8.8% respectively) was of the same order as reported in other studies using the same definitions (13.1-20.3% and 13.5% respectively) [5,6].

Other drugs known to be associated with constipation (such as iron, codein (opiates), calcium channel blockers, anticholenergic drugs, anticonvulsant drugs, anti-parkinson drugs, and antipsychotics) and diarrhoea (such as antibiotics, NSAID, psycholeptics, selective serotonin reuptake inhibitors and antihypertensives) did not show significant associations with constipation and diarrhoea in this study [3,7,16,18]. Some of them were significantly associated in the bivariate analyses (table 2) but not in the multivariable analyses. It shows the importance of pragmatic studies in the general population, and that such studies differ from traditional clinical trials. Reasons could be dose reduction or reduced compliance when ADRs occur, waning symptoms during long-term treatment, switching to other drugs without ADR, or a type II error.

Other predictors for constipation and diarrhoea were overall in accordance with other reports. There was a predominance of females with constipation and males with diarrhoea [4,5,7,16]. Constipation increased with age and seemed to be related to inactivity, coronary disease and neurological diseases [4,7,16,32]. The association between diarrhoea and mood disorders has been reported in other studies, whereas the associations to low education and osteoporosis remain unexplained [33].

### Strengths and weaknesses

Unlike most reports on ADRs, this study describes associations between drugs and symptoms related to everyday use of drugs in the general population. The design avoids problems related to clinical trials and surveys in selected, often elderly, populations. Except for the possibility that "trained complainers" were more prone to respond, there was no selection bias or loss to follow up, compliance and reporting of complaints were unaffected by the personnel's interest in the reports, and the treatment duration was probably longer than in most clinical trials. It is not known how the drugs were used, and incorrect use might increase ADRs. However, these results reflect associations related to everyday use of drugs, used correctly or not, but the cross sectional design gives no information about causality.

The ATC-classification of drugs used in this study relates ADRs to specific chemical compounds or groups with similar chemical compounds, which is a strength [23]. Most studies relate ADRs to broader and poorly defined groups of drugs e.g. antipsychotics and antihistamines, which are groups containing drugs with different ADRs, and therefore give insufficient information about ADRs related to each compound.

In addition to the commonly reported OR, we present the estimated changes in prevalence of the complaints when non-users start and users stop treatment. Unlike in randomized controlled trials, the distribution of covariates in subjects using and not using the drug differs. These risk differences are estimated taking into account differences in background variables among users and non users of the drug, as well as uncertainties in the estimates for the background variables [25]. This is a clinically more meaningful measure than OR.

The low response rate, 42% of all invited and 75% of the participants in the health survey might reduce the external validity. However, analyses of a similar study conducted by Norwegian Institute of Public Health in 2001 with a response rate of 46% showed no impact of the low response rate on self-selection [34].

The size of the study allows only demonstration of common ADRs. Common ADRs are defined by the authorities as prevalence rates between 1-10% [31]. The power of the study was 80% to detect an adverse event with a prevalence of 1%, given that 5% of the participants use the drug (α = 0.05). Clinically relevant information about individual drugs might have been missed since most drugs were used by less than 1% of the population.

The temporal relationship between drugs and symptoms in this study is uncertain. The questionnaire asked for symptoms last 3 months and use of drugs last 4 weeks. However, the temporal relationship is probably a minor problem since symptoms according to the Rome criteria are long lasting and regularly used drugs are most often long-term treatment.

Because the participants were asked for regularly used drugs the last four weeks, information about compliance, over the counter drugs, drugs taken to relieve symptoms, drugs taken on demand, general life style and food habits are insufficient. Analgesics (e.g. with codein and acetylsalicylic acid) and other drugs that influence on gastrointestinal function might therefore have been left out, and irregular and therefore not registered intake of laxatives and anti-diarrhoeas might have reduced the prevalence of the complaints. Therefore, the associations between drugs and the complaints do not prove, but only indicate causality.

## Conclusions

Everyday use of drugs was associated with an increased prevalence of constipation and diarrhoea, and polypharmacy with an additional risk of diarrhoea in the general population. Furosemide, levothyroxine sodium and ibuprofen were significantly associated with constipation, and carbamazepine and lithium with diarrhoea. The associations do not prove, but indicate that constipation and diarrhoea are common ADRs. In patients with constipation or diarrhoea, drug induced symptoms need to be considered and changes in drug regimens performed along with other interventions.

## Competing interests

The authors declare that they have no competing interests.

## Authors' contributions

GSF has worked up the data file, performed the statistical analyses under supervision of the medical statistician, interpreted the results, and written the manuscript. SL is responsible for the medical statistics and has participated in interpretation of the results and preparation of the manuscript. PGF prepared the questionnaire and parts the survey in collaboration with the Norwegian Institute of Public Health, is responsible for conception and design, and has participated in the statistical analyses and preparation of the manuscript and is the main supervisor of the project. All authors have read and approved the last version of the manuscript.

## Pre-publication history

The pre-publication history for this paper can be accessed here:

http://www.biomedcentral.com/1472-6904/11/2/prepub
